# Cigarette smoking and smoking-attributable diseases among Estonian physicians: a cross-sectional study

**DOI:** 10.1186/s12889-018-5105-6

**Published:** 2018-01-30

**Authors:** Mait Raag, Kersti Pärna

**Affiliations:** Institute of Family Medicine and Public Health, University of Tartu, Tartu, Estonia

**Keywords:** Physicians, Tobacco smoking, Attributable risk, Ischaemic heart disease, Chronic bronchitis, Lung emphysema, Chronic obstructive pulmonary disease, Estonia

## Abstract

**Background:**

Smoking is a risk factor for several diseases. Physicians are role models for their patients. Physicians who smoke underestimate the health risks of smoking and may be less likely to offer advice to help their patients to quit. The aim of this study was to: provide an overview of smoking behaviour among Estonian physicians; assess the relationship between smoking and ischaemic heart disease (IHD), chronic bronchitis (CB), and lung emphysema (LE); and estimate fractions of prevalences of the three diseases attributable to smoking.

**Methods:**

Self-administered questionnaires were sent to practising physicians (*n* = 5666) in Estonia in 2014. Prevalence of smoking and relative risks for IHD, CB and LE as well as the risks of IHD, CB and LE attributable to smoking were calculated by age and sex. Post-stratification was used to compensate non-response.

**Results:**

There were 535 male and 2404 female physicians participating. The prevalence of daily smoking was 12.4% (95% CI 10.4–14.4%) among men and 5.0% (95% CI 4.4–5.6%) among women. Mean duration of smoking among male and female daily smokers was 28.6 (95% CI 26.1–31.1) and 28.6 (95% CI 27.1–30.2) years. Compared to lifelong non-smokers, the age-adjusted risk for IHD was 1.29 times (95% CI 0.88–1.89) higher for men, but 1.69 times (95% CI 1.17–2.40) lower for all women who have ever smoked. The risk for CB was 2.29 (95% CI 1.30–4.03) times higher for smokers among men and, 1.32 (95% CI 0.95–1.82) among women; the risk ratio for LE was 4.92 (95% CI 1.14–21.1) among men and 2.45 (95% CI 0.63–9.52) among women. The smoking-attributable risk for IHD was 3.2% (95% CI 2.3–4.1%) among men and − 0.1% (95% CI -0.7–0.4%) among women; for CB 6.9% (95% CI 6.0–7.8%) and 4.2% (95% CI 3.5–4.8%); and for LE 18.8% (95% CI 17.0–22.5%) and 22.6% (95% CI 18.5–26.9%), respectively.

**Conclusion:**

Prevalence of daily smoking was relatively low among Estonian physicians (and twice lower among female physicians). The risk attributable to smoking was higher for LE and CB than for IHD.

**Electronic supplementary material:**

The online version of this article (10.1186/s12889-018-5105-6) contains supplementary material, which is available to authorized users.

## Background

Tobacco smoking is a major risk factor for cardiovascular diseases [1–3], including ischaemic heart disease (IHD) [4–6], and for pulmonary diseases, including chronic obstructive pulmonary disease (COPD) which is usually preceded by chronic bronchitis (CB) and is often manifested as obstructive bronchitis with lung emphysema (LE) [7, 8]. Effective tobacco control reduces the prevalence of smoking which logically results in lower incidences of the related diseases.

Physicians are widely regarded as health behaviour role models [9–11]. At the same time, physicians who smoke underestimate the health risks of smoking and may be less likely to offer advice to help their patients quit smoking [12–14].

Previous reports have shown that smoking prevalence among physicians in high income countries (e.g. the United Kingdom, the United States, Australia, and Finland) has decreased [15–18]. The prevalence of smoking among physicians was 4% in US in 1984 and 3% in Australia in 1996 already [15]. In Finland, the prevalence of smoking was 5% among male and 3% among female physicians in 2001 [19]. In Estonia, surveys concerning smoking among physicians have been carried out four times (1978, 1982, 2002, 2014), with smoking having decreased since 1982 (age standardized prevalence of daily smoking has decreased from 19 to 7%) [20].

Smoking prevalence in the total population in developed countries (including Estonia) has decreased during recent decades as well [21]. In Estonia, daily smoking among the general adult population (16–64-years olds) decreased from 50% in 1994 to 31% in 2014 among men and from 21 to 16% among women [22, 23]. As it has been suggested that the decrease in smoking among doctors is followed by the decrease in smoking in the general population [24, 25], it is beneficial to monitor physicians’ smoking habits to understand the current and future tobacco epidemic situations in the region.

Many studies have shown that smoking increases the risk of heart and lung diseases [1–6]. In 2015, diseases attributable to smoking had the second highest ranking among all of the diseases burdening people globally [26]. Measured in disability-adjusted life years (DALYs), tobacco smoke was associated with 24% of DALYs caused by IHD and 44% of DALYs caused by COPD [27]. However, the risk of a disease that is attributable to smoking depends both on the prevalence of smoking and the strength of the association between smoking and the occurrence of the disease. For example, in 2010 the prevalence of daily smoking (in 15-year olds or above) was 23% in Ukraine and Italy and 39% in Albania [21], but 59% of DALYs caused by COPD were associated with tobacco smoke in Ukraine, 62% in Albania and 71% in Italy [27]. Therefore, if calculated for one population, the smoking-related burden on health is not easily conveyable to another.

To our knowledge no-one has estimated the proportions of prevalence of IHD, CB and LE attributable to smoking before, neither among physicians nor among the general population in Estonia. The aim of this paper was to provide an overview of smoking behaviour among Estonian physicians; assess the relationship between smoking and IHD, CB, and LE; and estimate fractions of prevalences of the three diseases attributable to smoking.

## Methods

### Study conduct

We conducted a cross-sectional study with total sampling, the conduct of which is described in detail elsewhere [28]. All physicians working in Estonia are registered in the Estonian Health Care Professionals Registry [29]. The registry was queried for practising physicians in July 2014 and the data was linked with the population registry to obtain the physicians’ home addresses. In October 2014, study materials were sent to all physicians with a known home address (*n* = 5666). The four-page questionnaire was similar to that which was used in the previous 2002 study on smoking among Estonian physicians, which had been adapted from a questionnaire originally developed by the World Health Organization [30]. A reminder letter was sent to those who had not responded within 4 weeks, and study materials were sent again to those who still had not responded in the end of November 2014. The responses were accepted until March 2015.

### Data on smoking behaviour and smoking related diseases

The participants were asked if they had ever been smoking regularly at least for a year; their age of taking up smoking; and their current smoking behaviour. These questions allowed to define four categories: never smokers (who answered ‘no’ to the question asking about ever smoking regularly), ex-smokers (who answered ‘yes’ to the question asking about ever smoking regularly and ‘no’ to the question regarding current smoking), occasional smokers (who answered ‘yes’ to the question regarding current smoking and ‘no’ to the question regarding daily current smoking), daily smokers (who answered ‘yes’ to the question asking about daily current smoking).

Daily smokers were asked the number of cigarettes they smoked each day (up to 10, 11–20, 21–30, or more than 30); therefore, only the proportions of daily smokers who smoke more than a pack (20 cigarettes) in a day is described and this information is not considered in further analysis.

A multiple-choice question was asked about currently having IHD, CB, or LE; refusal to answer was an additional option. Anyone who did not choose from any of the options (including refusal to answer) was considered to be a person who does not have any of the afflictions.

### Statistical analysis

To reduce bias caused by differential response rate (i.e. younger people and men being under-represented) post-stratification weights (based on sex and 5-year age groups) were used to compensate unit non-response (non-returned or unfilled questionnaires) [31]. Unadjusted and age-adjusted risk ratios (RR) were estimated by log-binomial regression. Classical statistical methods assume infinite population, but as about a half of all physicians in Estonia participated in the study, finite population correction [31] increasing the precision of the estimates was applied to calculate Wald-type 95% confidence intervals (CI). Agresti-Coull 95% confidence intervals with finite population correction were calculated for zero proportions, based on the weighted estimate of the total size of the respective subpopulation. To account for multilevel exposure (four smoking categories), level-specific adjusted attributable risks (AR) were calculated, defined as

$$ {AR}_k=\sum \limits_s\frac{\left[P\left(D|{E}_k,{C}_s\right)-P\left(D|{E}_0,{C}_s\right)\right]P\left({E}_k|{C}_s\right)P\left({C}_s\right)}{P(D)}, $$

where *P*(*D*| *E*_*k*_, *C*_*s*_) is the proportion of diseased among individuals with exposure level *k* (0 = never smoker) and confounder level *s*, *P*(*E*_*k*_| *C*_*s*_) is the prevalence of exposure level *k* among individuals with confounder level *s*, *P*(*C*_*s*_) is the prevalence of confounder level *s*, and *P*(*D*) is the prevalence of disease [32]. The overall risk attributable to exposure to smoking was estimated by case-load method:

$$ AR=\sum \limits_k{w}_k{AR}_k, $$

where *AR*_*k*_ denotes AR specific to exposure level *k* and *w*_*k*_ equals the proportion of cases in level *k* [33]. To account for finite population, 95% confidence intervals for AR were estimated using the replication bootstrap method [34]. Age was grouped to four groups based roughly on the quartiles in the target population. To adjust for years smoked, exposure was defined by the combination of smoking status and years smoked (0–5, 6–15, 16–25, > 25 years).

EpiInfo 3.5.3 [35] was used for double data entry. Statistical environment R 3.2.2 [36] with survey package [37] was used for the calculations.

## Results

### Target population and study participants

In total, 2939 practising physicians (535 men and 2404 women) participated in this study. The crude response rate was 52%, while the corrected response rate (excluding physicians who were unavailable, retired, had an incorrect address, had left Estonia, or had died) was 53%. About four fifths (79,6%) of the respondents lived in cities or towns.

The age of the study participants was between 24 and 86. Men (18% in the sample vs. 23% in the population) as well as younger physicians (mean age 51.7 years in the sample vs. 51.2 in the population) were slightly under-represented in the sample. The distribution of physicians by age groups and sex in the target population and the proportions participating in the study in each age-sex group is given in Table 1. Counts of physicians by sex, age, smoking status, years smoked, presence of IHD, CB and LE weighted by age and sex are given in Additional file 1.

**Table 1:** Age-sex distribution of the target population and study participants of the survey among physicians in Estonia

	Target population (% of all 5666 working physicians in Estonia)			Participants (% of age-sex group in the target population)		
Age	Male	Female	Total	Male	Female	Total
≤ 40	330 (6%)	1069 (19%)	1399 (25%)	123 (37%)	586 (55%)	709 (51%)
41–50	279 (5%)	924 (16%)	1203 (21%)	118 (42%)	492 (53%)	610 (51%)
51–60	344 (6%)	1190 (21%)	1534 (27%)	125 (36%)	641 (54%)	766 (50%)
≥ 61	330 (6%)	1200 (21%)	1530 (27%)	170 (52%)	684 (57%)	854 (56%)
Total	1283 (23%)	4383 (77%)	5666 (100%)	536 (42%)	2403 (55%)	2939 (52%)

### Prevalence and duration of smoking

Current smokers (occasional and daily combined) constituted 8.4% (95% CI 7.6–9.1%) of Estonian physicians. The prevalence of daily smoking was highest in the age group of 51–60 both among men and women (Table 2). Among male physicians 53.0% had never smoked, 31.5% had quit smoking, 3.1% smoked occasionally, and 12.4% smoked daily. The respective proportions among female physicians were 74.5, 19.2, 1.3, and 5.0%. Among male daily smokers 11.4% (95% CI 6.9–18.0%) smoked at least 21 cigarettes per day, while among female daily smokers this proportion was 7.7% (95% CI 4.9–12.0%).

**Table 2:** Prevalence of smoking (95% CI) by sex and age group among physicians in Estonia (population estimates)

Sex	Age	Never smokers	Ex-smokers	Occasional smokers	Daily smokers
Male	≤ 40	74.9 (69.6–80.2)	12.7 (8.7–16.8)	3.2 (1.0–5.3)	9.2 (5.6–12.8)
	41–50	52.6 (46.4–58.9)	35.9 (29.8–41.9)	1.7 (0.1–3.4)	9.8 (6.1–13.5)
	51–60	41.5 (35.5–47.5)	35.2 (29.4–41.0)	7.2 (4.1–10.3)	16.1 (11.6–20.5)
	≥ 61	43.5 (38.2–48.9)	42.3 (37.0–47.7)	0.0 (0.0–2.2)	14.1 (10.2–18.0)
	Total	53.0 (50.0–56.0)	31.5 (28.7–34.2)	3.1 (2.0–4.2)	12.4 (10.4–14.4)
Female	≤ 40	85.0 (83.0–87.0)	11.1 (9.3–12.9)	1.5 (0.8–2.2)	2.4 (1.6–3.3)
	41–50	77.5 (75.0–80.1)	17.0 (14.7–19.3)	2.0 (1.2–2.9)	3.5 (2.4–4.6)
	51–60	66.8 (64.3–69.3)	24.1 (21.8–26.4)	1.1 (0.5–1.7)	8.0 (6.5–9.4)
	≥ 61	70.5 (68.1–72.9)	23.2 (21.0–25.4)	0.7 (0.3–1.2)	5.6 (4.4–6.8)
	Total	74.5 (73.3–75.7)	19.2 (18.1–20.3)	1.3 (1.0–1.6)	5.0 (4.4–5.6)
Total		69.7 (68.5–70.9)	21.9 (20.9–23.0)	1.7 (1.4–2.0)	6.7 (6.0–7.3)

In general, smoking behaviour by age groups was somewhat different between men and women (Table 2). Among physicians younger than 41 the prevalence of lifelong non-smoking was 10% higher in women compared to men, whereas among physicians older than 40 the difference was at least 25%. In the youngest age group (≤ 40 years), the proportion of ex-smokers was almost the same among men (12.7%) compared to women (11.1%), but not in the older age groups (the difference between men and women was > 10%). The proportion of daily smokers ranged from 9% to 16% among men, but from 2% to 8% among women in all age groups.

In general, men had longer history of smoking than women (Table 3). Male ex-smokers had smoked 16.6 years, occasional smokers 17.1, and daily smokers 28.6 years on average. The corresponding numbers among women were 11.3, 14.4, and 28.6 years.

**Table 3:** Average duration of smoking in years (95% CI) by sex and age group among physicians in Estonia (population estimates)

Sex	Age	Ex-smokers	Occasional smokers	Daily smokers
Male	≤ 40	8.1 (6.6–9.6)	12.2 (10.7–13.8)	9.7 (7.8–11.6)
	41–50	15.1 (13.5–16.6)	15.5 (5.5–25.5)	22.5 (19.7–25.4)
	51–60	14.9 (12.9–16.9)	19.4 (13.9–25.0)	30.0 (26.4–33.7)
	≥ 61	21.8 (19.6–24.0)	-^a^	43.1 (40.5–45.6)
	Total	16.6 (15.4–17.7)	17.1 (13.3–20.9)	28.6 (26.1–31.1)
Female	≤ 40	5.5 (4.8–6.2)	7.4 (5.2–9.5)	13.0 (11.0–15.0)
	41–50	9.2 (8.2–10.2)	17.9 (14.0–21.9)	22.3 (20.0–24.6)
	51–60	11.3 (10.3–12.4)	11.3 (6.1–16.5)	28.4 (26.5–30.3)
	≥ 61	14.8 (13.5–16.1)	26.1 (16.0–36.2)	38.2 (35.6–40.8)
	Total	11.3 (10.7–11.9)	14.4 (11.6–17.1)	28.6 (27.1–30.2)
Total		13.0 (12.4–13.6)	15.5 (13.2–17.8)	28.6 (27.2–30.0)

^a^Among the participants there were no men over 60 smoking occasionally

### Prevalence and relative risk of IHD, CB, and LE

There were 762 physicians (25.9% of the sample) who did not wish to respond to the diseases-related questions. Among the rest (*n* = 2177), there were 122 IHD cases (77 among never smokers), 106 CB (55 among never smokers), and 11 LE cases (3 among never smokers).

In general, the risk of these three diseases was lowest among never smokers. Overall IHD prevalence was 5.3% (95% CI 4.7–6.0%); 4.5% (95% CI 3.8–5.2%) among never, 7.0% (95% CI 5.3–8.6%) among ex-, 7.4% (95% 0.6–14.2%) among occasional, and 7.0 (95% CI 3.9–10.2%) among daily smokers. Overall CB prevalence was 4.8% (95% CI 4.2–5.4%); 3.4% (95% CI 2.8–4.0%) among never, 5.9% (95% CI 4.4–7.4%) among ex-, 3.8% (95% CI 0.0–8.9%) among occasional, and 17.7% (95% CI 12.8–22.6%) among daily smokers. Overall LE prevalence was 0.6% (95% CI 0.4–0.8%); 0.2% (95% CI 0.0–0.3%) among never, 1.6% (95% CI 0.8–2.5%) among ex-, 0% (95% CI 0.0–0.0%) among occasional, and 1.2% (95% CI 0.0–2.8%) among daily smokers.

**Fig. 1:**
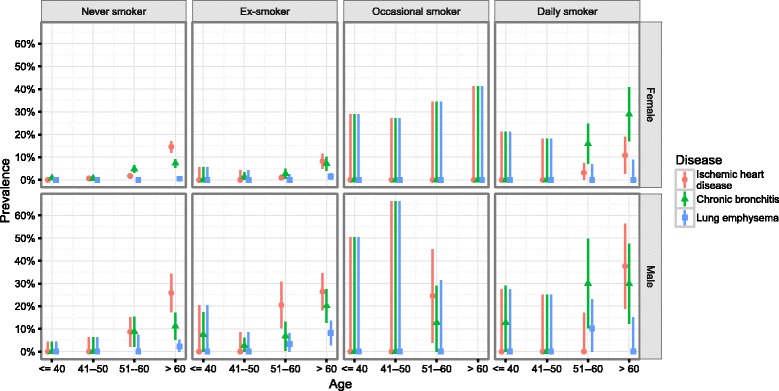
Prevalence (95% CI) of ischaemic heart disease, chronic bronchitis and lung emphysema by sex and age groups among physicians in Estonia (population estimates)

There was a clear trend of increasing prevalence of IHD and CB by age groups, the trend for LE prevalence was less visible due to a small number of cases (Fig. 1). Illustrating the effect of smoking, those trends were more pronounced among daily smokers compared to lifelong non-smokers. IHD was clearly more prevalent among men than women.

Relative risks for the three diseases comparing smokers to non-smokers were higher among men than among women. Compared to lifelong non-smokers, the age-adjusted risk for IHD was 1.29 times (95% CI 0.88–1.89) higher for all men who have ever smoked, but 1.69 times (95% CI 1.17–2.40) lower for all women who have ever smoked. Age-adjusted risk ratio for CB comparing ever smokers to never smokers was 2.29 (95% CI 1.30–4.03) among men and 1.32 (95% CI 0.95–1.82) among women; respective risk ratios for LE were 4.92 (95% CI 1.14–21.1) and 2.45 (95% CI 0.63–9.52). Age-adjusted RR having any of the three conditions comparing those that have smoked to never smokers was 1.66 (95% CI 1.23–2.25) for men and 0.89 (95% CI 0.70–1.12) for women.

There was a clear association between the number of years smoked and the risk of IHD or CB, especially among men (Table 4). Compared to those men who had been smoking up to 5 years, the age-adjusted risk of any of the three conditions was 1.04 times higher for those men who had been smoking for 6–15 years, 1.68 times higher for those who had been smoking for 16–25 years, and 2.46 times higher for those who had been smoking for more than 25 years. The corresponding age-adjusted risk ratios for women were 2.56, 2.36, and 7.11.

**Table 4:** Age-adjusted relative risk* (95% CI) for ischaemic heart disease (IHD) and chronic bronchitis (CB) by years smoked among physicians who have ever smoked, compared to those who have smoked up to 5 years (population estimates)^a^

Condition^b^	Sex	Ex-smokers			Ever smoked^c^		
		Years smoked			Years smoked		
		6–15	16–25	>25	6–15	16–25	>25
IHD	Male	1.82 (0.61–5.41)	2.72 (0.92–7.97)	*3.02 (1.08–8.45)*	2.44 (0.85–7.02)	*3.27 (1.15–9.32)*	*2.91 (1.08–7.83)*
	Female	1.83 (0.60–5.56)	**	*5.40 (2.04–14.28)*	1.75 (0.56–5.42)	**	*4.32 (1.59–11.75)*
	Total	2.17 (0.96–4.89)	1.92 (0.77–4.82)	*5.70 (2.65–12.26)*	*2.38 (1.07–5.30)*	2.10 (0.88–5.01)	*4.54 (2.19–9.40)*
CB	Male	1.08 (0.31–3.74)	2.04 (0.60–6.97)	2.31 (0.73–7.31)	0.79 (0.30–2.06)	1.17 (0.42–3.25)	2.10 (0.86–5.16)
	Female	3.61 (0.80–16.30)	*4.95 (1.05–23.38)*	*5.75 (1.14–29.04)*	*4.64 (1.05–20.47)*	*6.75 (1.56–29.16)*	*12.38 (2.97–51.61)*
	Total	2.22 (0.85–5.82)	*3.55 (1.32–9.55)*	*4.86 (1.86–12.72)*	1.91 (0.85–4.32)	*2.77 (1.20–6.38)*	*5.42 (2.49–11.78)*
Any condition^d^	Male	1.02 (0.45–2.33)	1.91 (0.87–4.17)	*2.65 (1.31–5.36)*	1.04 (0.51–2.12)	1.68 (0.85–3.33)	*2.46 (1.35–4.50)*
	Female	2.20 (0.90–5.39)	1.70 (0.60–4.81)	*6.59 (2.83–15.33)*	2.56 (1.03–6.20)	2.36 (0.91–6.10)	*7.11 (3.13–16.14)*
	Total	1.76 (0.93–3.30)	*2.30 (1.19–4.44)*	*7.11 (3.13–16.14)*	*1.79 (1.01–3.18)*	*2.23 (1.24–4.03)*	*5.13 (3.06–8.62)*

^a^ Age-adjusted risk ratios among occasional and daily smokers were not estimable

^b^ Age-adjusted risk ratios for lung emphysema were not estimable

^c^ Including ex-, occasional, and daily smokers

^d^ Including ischaemic heart disease, chronic bronchitis, and lung emphysema

* Statistically significant risk ratios (p < 0.05) are italicized

** Not estimable

### Population attributable risk

After adjusting for age and years smoked, the prevalence of LE attributable to smoking was 19% among men and 23% among women, while the attributable prevalences for IHD and CB were much lower (3 and 7% for men, 0 and 4% for women, respectively) (Table 5). While the difference between unadjusted and adjusted AR was 17% for IHD and 17% for CB among men, the respective differences were much smaller among women (3 and 4%).

**Table 5:** Smoking-attributable risk (AR) of ischaemic heart disease (IHD), chronic bronchitis (CB), and lung emphysema (LE) among Estonian physicians attributable to smoking (%), with 95% CI (population estimates)

Sex	Condition	Unadjusted AR	AR adjusted for age	AR adjusted for age and years smoked
Male	IHD	20.6 (18.0–23.2)	8.6 (5.9–11.3)	3.2 (2.3–4.1)
	CB	24.2 (22.3–26.5)	17.5 (15.3–20.1)	6.9 (6.0–7.8)
	LE	53.4 (47.8–61.8)	47.9 (41.9–57.6)	18.8 (17.0–22.5)
	Any above	20.6 (19.2–22.2)	11.8 (10.3–13.3)	5.2 (4.7–5.8)
Female	IHD	-3.0 (−4.2–-2.1)	-9.7 (-10.8–-8.7)	-0.1 (-0.7–0.4)
	CB	8.1 (7.0–9.0)	4.8 (3.6–5.8)	4.2 (3.5–4.8)
	LE	40.9 (30.7–50.0)	35.5 (24.3–45.4)	22.6 (18.5–26.9)
	Any above	2.1 (1.3–2.7)	-2.7 (-3.5…-2.0)	1.0 (0.7–1.3)

## Discussion

This study focused on smoking behaviour and the relationship between smoking and IHD, CB, and LE among Estonian physicians. The years smoked were associated with the risk for these three diseases. The risk attributable to smoking was higher for pulmonary diseases than for ischaemic heart disease.

### Smoking behaviour

In Estonia 53% of male and 75% of female physicians had never smoked, while 12% of male and 5% of female physicians smoked daily in 2014. Smoking behaviour among younger (up to 40 years old) male and female physicians were more similar to each other than among physicians older than 40 years. Similar phenomenon has also been observed in other studies and has been associated with changing socio-economic circumstances [38]. Compared to the previous similar study from 2002 [30], the prevalence of daily smoking had decreased 1.5-fold (18% in 2002 vs. 12% in 2014) and lifelong non-smoking had increased 1.2-fold (43% in 2002 vs. 53% in 2014) among male physicians, but had remained about the same among female physicians (respective proportions were 6% in 2002 vs. 7% in 2014, and 74% in 2002 vs. 70% in 2014). This is in line with trends in the general adult population where smoking among men has decreased faster than among women [23, 39]. Compared to the 16–64-year old general adult population in Estonia, the prevalence of physicians who have never smoked in this study was considerably higher (by 25% among men and 22% among women) and the prevalence of daily smokers considerably lower (by 19% among men and by 11% among women), but age-related differences correlate to those of the general population [23]. At the same time the prevalence of smoking among physicians in Estonia in 2014 was comparable with smoking among physicians in the United States and Australia in the 1980s [16, 40]. Thus, the prevalence of smoking among physicians in Estonia is still higher than in many developed countries. However, smoking prevalence among physicians in some developed countries like France, Italy and Japan has remained high with the prevalence of current smoking over 25% [18, 41, 42].

Our results support the hypothesis that the decline of smoking among the general population follows the decline of smoking among physicians [24, 25]. While daily smoking has decreased among Estonian physicians at least since 1982 [20], it had its peak in the general population in 1994 when the prevalence of daily smoking was 50% among men and 21% among women [22]. Since 1994, the prevalence of daily smoking has decreased among the general population in Estonia, especially among men [21].

Interestingly, in the current study women aged between 41 and 50 years who smoked occasionally had been smoking longer time (17.9 years in average) than women aged between 51 and 60 (11.3 years). In addition to recall bias, this difference, although statistically not significant at the 0.05 significance level (results omitted), can be partially explained by the average age they started smoking. We found that women in the age group of 41–50 years started smoking younger than women in the age group of 51–60. However, the time between starting smoking and the study conduct was generally longer than the number of years smoked that the subjects reported. This indicates that the subjects had had smoke-free periods. Hence, it is possible that women in the age group of 51–60 years had had more and/or longer breaks in smoking, although these data were not collected in our study.

### Smoking attributable diseases

Although the prevalence of IHD and CB was similar among male and female non-smokers (7 and 4% among men, 4 and 3% among women), this prevalence was much higher among formerly or currently smoking men, compared to women of the same age and smoking class. This can be partially explained by the number of cigarettes smoked daily, which is higher among men in many countries [43]. Although in our study it was the similar case among daily smokers, data on the number of cigarettes smoked was not collected from occasional and ex-smokers, inhibiting to account for the intensity of smoking. Additionally, prevalence of other IHD risk factors (e.g. excess drinking) are known to be higher among men than among women in Estonia [44], but these data were not collected in our study.

Unsurprisingly, there was a clear association between the risk of smoking-related diseases and years smoked: age-adjusted risk for the IHD and CB was higher among those men and women who had smoked longer. These results are consistent with worldwide literature [1, 2]. Although the prevalence of LE was low (0.6%), it is noteworthy that the majority of cases were among ex- and current smokers.

In our study, the effect of smoking was clearest in the prevalence of CB. It was reported that for current smokers in the United States, CB was the most prevalent condition, followed by LE [45]. While tobacco smoking is the most prominent risk factor for pulmonary diseases [27], there are many additional strong risk factors for cardiovascular diseases (e.g. excessive alcohol consumption, sedentary lifestyle) [46]. However, data on those risk factors was not collected in our study.

It has been indirectly estimated that in 2015 in Estonia, 8% of deaths caused by IHD among women and 22% among men in general population were associated with tobacco smoke (corresponding proportions being similar for years of life lost and years of life lived with a disability) [27]. In our study the unadjusted attributable risks were in comparable ranges for both men (21%) and women (about 0%). Although mortality and prevalence are directly not comparable, this similarity in the measures of smoking-attributable burden of IHD in the general population compared to physicians is not surprising, as IHD has many strong risk factors besides smoking [46].

One might assume that due to cleaner working environment of physicians compared to that of an average citizen’s, among physicians other risk factors (especially air pollution) would not contribute to the risk of pulmonary diseases as much as smoking would do. However, 80% of deaths caused by COPD among men and 41% among women were associated with tobacco smoke in the general population (and these proportions were similar for other measures of burden of disease) [27] while in our study the unadjusted attributable risks for pulmonary diseases were generally lower (24% of CB among men, 8% among women; 53% of LE among men, 41% among women). Although it can partially be explained by the fact that more physicians live in cities or towns compared to the general adult population (63%) [47], this finding was not expected and warrants further investigation.

### Strengths and weaknesses

Consisting of working physicians, the study population can be considered relatively homogeneous, which reduces the effect of possible confounders (e.g. education, income). Almost half of the target population participated in the study, resulting in more precise estimates. Our sample had a definite frame (Estonian Heath Care Professionals Registry) with the information on the age and sex of physicians which allowed us to compensate for non-response by post-stratification.

There were some physicians who contacted the study team asking whether they should participate if they are non-smokers. Therefore, non-smokers might be under-represented in the final sample. On the other hand, it is plausible that subjects who behave in a socially less desirable way (e.g. smoke daily) were less inclined to participate in this study. Physicians are perceived as most knowledgeable about the devastating effects of smoking; therefore, they may be prone to self-deception or understatement. However, the anonymity of the questionnaire supports the possibility of the responses being true. As smoking behaviour as well as health are associated with age and sex, we believe that post-stratification helped us to reduce that bias.

The questionnaire was self-administered, which may have introduced some information bias. For example, the number of years smoked might have been reported imprecisely. Considering the IHD, CB and LE, however, physicians probably have better knowledge about their health condition compared to a layman, which makes the data on self-reported health condition reasonably reliable. Nevertheless, a considerable number of physicians chose not to reveal data on having the three diseases we studied. In addition, we were not able to make certain that those who did not choose from any of the multiple choices regarding smoking-related health condition (including the option to refuse to answer) were free from those conditions. Therefore, the prevalence of IHD, CB, and LE might be underestimated. Hence it is plausible that the attributable risk estimates are underestimates as well.

Many factors may have influenced the response rate [48]. For example, physicians on sick leave or on holiday might have had more time to fill in the questionnaire. We did not study the reasons of non-response in this survey; therefore, the possible bias introduced by differential response rates is difficult to assess. However, we believe that the long response period (6 months) and the length of the questionnaire (four pages) has substantially decreased the risk of this bias. Additionally, the age and sex distribution of the population, often being strongly correlated with such possible factors (e.g. workload), was taken into account by post-stratification which has decreased this possible bias even more.

The cross-sectional design of this study was suitable for estimating prevalences. Although we do not know the timing of events (taking up smoking, quitting, disease occurrence), which inhibits causal interpretation of attributable risks, it has been observed that people more often quit than start smoking after getting pulmonary [49] or cardiovascular diseases [50]. Therefore, it is safe to assume that among ex- and current smokers IHD, CB, or LE occurred after being exposed to smoking.

## Conclusions

Prevalence of daily smoking was relatively low among Estonian physicians being twice lower among female physicians compared to male. Risk attributable to smoking was higher for lung emphysema and chronic bronchitis than for ischaemic heart disease, hence communicating the information about the negative effects of smoking to the people who already are at greater risk for pulmonary diseases is strongly advised.

Tobacco control policies should focus on increasing smokers’ willingness to quit smoking and providing the necessary support and therapies. This would increase the likelihood of successful smoking cessation among physicians which would have major benefits to health of the general population. Further studies are required to continue monitoring the smoking behaviour among Estonian physicians.

## Additional file

Additional file 1: Weighted counts. Weighted counts of study participants by sex, age, smoking status, smoking duration, presence of ischaemic heart disease, lung emphysema and chronic bronchitis. (XLSX 129 kb)

## Abbreviations

AR: Attributable risk
CB: Chronic bronchitis
CI: Confidence interval
COPD: Chronic obstructive pulmonary disease
IHD: Ischaemic heart disease
LE: Lung emphysema
RR: Relative risk
